# Unraveling the Chloroplast Genomes of Two *Prosopis* Species to Identify Its Genomic Information, Comparative Analyses and Phylogenetic Relationship

**DOI:** 10.3390/ijms21093280

**Published:** 2020-05-06

**Authors:** Sajjad Asaf, Abdul Latif Khan, Arif Khan, Ahmed Al-Harrasi

**Affiliations:** 1 Natural and Medical Sciences Research Center, University of Nizwa, Nizwa 616, Oman; sajadasif2000@gmail.com; 2 Genomics Group, Faculty of Biosciences and Aquaculture, Nord University, 8049 Bodø, Norway; arif.biotec@gmail.com

**Keywords:** plastid genome, Fabaceae, phylogenetic position, comparative analysis, inverted repeats

## Abstract

Genus *Prosopis* (family Fabaceae) are shrubby trees, native to arid and semi-arid regions of Asia, Africa, and America and known for nitrogen fixation. Here, we have sequenced the complete chloroplast (cp) genomes of two *Prosopis* species (*P*. *juliflora* and *P*. *cineraria*) and compared them with previously sequenced *P. glandulosa*, *Adenanthera microsperma*, and *Parkia javanica* belonging to the same family. The complete genome sequences of *Prosopis* species and related species ranged from 159,389 bp (*A*. *microsperma*) to 163,677 bp (*P*. *cineraria*). The overall GC contents of the genomes were almost the similar (35.9–36.6%). The *P. juliflora* and *P. cineraria* genomes encoded 132 and 131 genes, respectively, whereas both the species comprised of 85 protein-coding genes higher than other compared species. About 140, 134, and 129 repeats were identified in *P*. *juliflora*, *P*. *cineraria* and *P*. *glandulosa* cp genomes, respectively. Similarly, the maximum number of simple sequence repeats were determined in *P*. *juliflora* (88), *P*. *cineraria* (84), and *P. glandulosa* (78). Moreover, complete cp genome comparison determined a high degree of sequence similarity among *P*. *juliflora*, *P. cineraria,* and *P. glandulosa,* however some divergence in the intergenic spacers of *A. microsperma* and *Parkia javanica* were observed. The phylogenetic analysis showed that *P*. *juliflora* is closer to *P*. *cineraria* than *P. glandulosa*.

## 1. Introduction

The *Prosopis* L. genus belongs to the Leguminosae (Fabaceae) family, sub-family Mimosoideae. *Prosopis* comprises about 44 species, classified into five sections: *Prosopis, Monilicarpa, Strombocarpa, Algarobia,* and *Anonychium* [1]. *Prosopis* genus can be found around the world in arid and semi-arid regions, including Central and North Africa, South and North America, and the Caribbean region. It has both economic and ecological reputation in arid regions [1,2] and is related with chaquenian areas in Brazil [3]. The genus is mostly pollinated by insects [1,4,5], which shows a short distance of pollen dispersion [4,5]. *Prosopis* taxa grow in most of the world’s warm arid and semi-arid areas, both as introduced and native species [6]. They have been introduced universally and have become naturalized or invasive in various part of the world [7].

*Prosopis cineraria* is one of the most common trees of the Indian desert and locally known as Khejri. This is an important agroforestry tree and a renewable source of timber, fodder and fuel used by local populations [8]. It is an important part of Asian desert ecosystems due to biomass production and it provides desert soil, provides greenery and help to fix atmospheric nitrogen [9,10]. On the other hand, *P. juliflora* is a xerophytic evergreen tree which can grow in different climatic conditions and on variety of soils [11]. *P. juliflora* can grow up to 14 m having an open canopy and a large crown. The taxonomy of *P. juliflora* remains unclear due to the morphological similarities with *P. pallida* which cause confusion in their identification [6]. Similarly, genus *Prosopis* phylogenetic relationships have been controversial for a long time and various studies have suggested taxonomic revision [12,13].

The chloroplast is a vital organelle that plays a central role in various important biochemical processes especially in plant photosynthesis [14]. Due to an abundance within plants, slow mutation rate, maternal inheritance, and relatively small genome size chloroplast DNA (cpDNA) has been extensively used in genetic studies [15]. The gene content and structure of cp genomes among angiosperms are well conserved but there are exceptions such as parasitic plants with a reduced gene set and loss of IR. Chloroplast genome comprise two inverted repeat (IR) sequences which separates a small single copy (SSC) and large single copy (LSC) region and complete cp genome range in size from 107 to 218 kb [16]. Cp genome has been extensively used to infer the evolutionary history of most angiosperms due to its maternally inherited nature. These features makes cp genome very useful in phylogenetic and phylogeographic studies, especially at higher taxonomic levels [17]. Similarly, recent research have verified that phylogenetic analysis based on whole genome data set and all shared protein-coding genes can deliver better phylogeographic and phylogenetic resolution [18,19]. Maternally-inherited markers are often better predictors of interspecific gene flow [20], resulting in shared genetic structure, rather than the detection of incomplete lineage sorting [21,22]. Chloroplast DNA analyses have enabled the advancement of strategies for the conservation of various angiosperm species [23].

The development of next generation sequencing (NGS) technologies have allowed for the sequencing of entire chloroplast genomes. Many chloroplast genomes of sub family Caesalpinioideae have already been determined by NGS [24,25]. Similarly, the quickly evolving loci recognized by numerous researchers are very significant and have more resolving power than traditional molecular markers to resolve ambiguous phylogenetic relationships [26,27]. Therefore, numerous researchers have concentrated to determine genic regions among specific genera or families to get valuable information about molecular markers for future studies [26,27,28].

Cp genome variation has opened new significant understandings into the domestication origins of various crops such as citrus and apple [29,30,31]. In the current study, the complete chloroplast genomes of *P. juliflora* and *P. cineraria* were sequenced and elucidated their structural organization and performed comparative analysis with the genomes of related species viz. *P. glandulosa*, *A. microsperma* and *P. javanica* cp genomes. Furthermore, we identified the simple sequence repeats (SSRs), dispersed repeat sequences, sequence divergence and phylogenetic relationship among these studied cp genomes.

## 2. Results

### 2.1. General Features and Organization of Chloroplast Genomes

**Figure 1:**
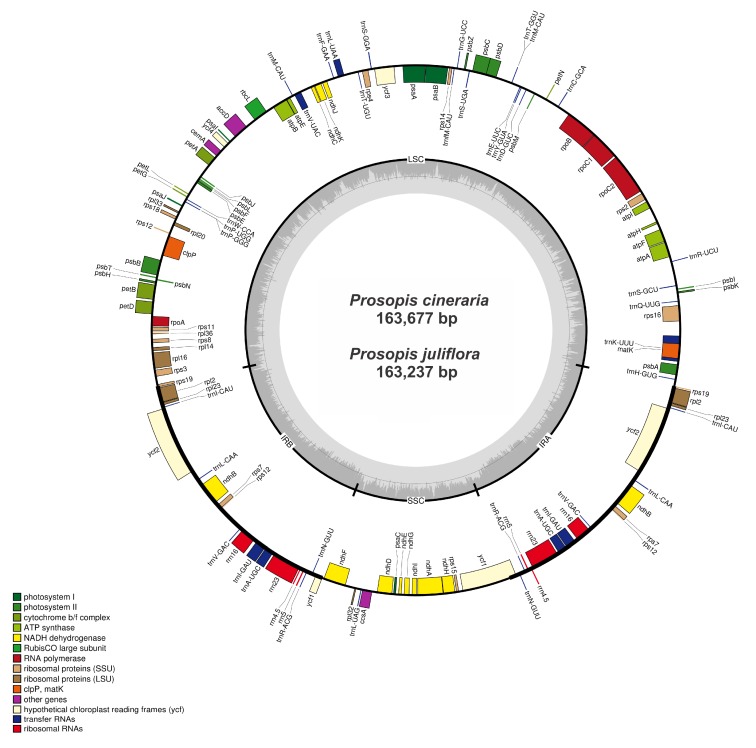
Genome map of the *P. juliflora* and *P. cineraria* cp genomes. The extent of the inverted repeat regions (IRs) is represented by thick lines, which divide the cp genome into large (LSC) and small (SSC) single copy regions. Genes drawn inside the circle are transcribed clockwise, while those outside of the circle are transcribed counter clockwise. Genes belonging to different functional groups are color coded. The dark grey in the inner circle corresponds to the GC content, while the light grey corresponds to the AT content.

The complete chloroplast genomes of the two sequenced *Prosopis* species, *Prosopis juliflora* (Pj) (MN104889) and *Prosopis cineraria* (Pc) (MN104890) are circular molecules like typical angiosperm cp genomes having quadripartite structures. The sizes of the *P. juliflora* and *P. cineraria* cp genomes are 163,237 and 163,677 bp, respectively (Figure 1 and Table 1). Both *P. juliflora* and *P. cineraria* cp genomes were analyzed and compared with three associated cp genomes, with sizes ranging from 159,389 bp (*A. microsperma*) to 163,677 bp (*P. cineraria*) (Table 1). Similar to earlier reported angiosperm cp genomes [32,33,34,35], the *P. juliflora* and *P. cineraria* cp genomes are typical circular in nature comprising of four parts: (a) LSC region of 92,495 bp and 92,937 bp, covering 56.6% and 56.7% in the genomes respectively; (b) SSC region of 18,880 bp and 18,878 bp, covering 11.5% and 11.53% in the genome; (c) two IR regions separating the SSC and LSC regions, which were 25,931 bp and 25,931 bp in size, covering the same amount 15.8% in both the genomes respectively. The *P. juliflora* and *P. cineraria* cp genomes encodes 132 and 131 genes respectively, including 85 protein-coding genes, 38 tRNA (*P. cineraria*) and 39 tRNA genes (*P. juliflora*), and 8 rRNA genes each (Figure 1). Among the annotated genes, 16 genes (*pet*D, *pet*B, *atp*F, *rpl*2, *rps*16, *rpl*16, *rpoC*1, *rps*12, *ndh*A, *ndh*B, *trn*I*-GAU, trn*A*-UGC, trn*K*-UUU, trn*L*-UAA, trnV-UAC,* and *trn*G*-GCC*) have one intron each, and two genes (*ycf*3, *clp*P) comprised two introns each (Table 2). The *rps12* gene was trans-spliced; the 5′ end exon was detected in the LSC region and the 3′ exon was observed in the IR region, as in most other angiosperms. The protein-coding genes included 12 genes encoding small ribosomal proteins (*rps*2*,* 3, 4, 7, 8, 11, 12, 14, 15, 16, 18, and 19), eight genes encoding large ribosomal proteins (*rpl*2, 14, 16, 20, 23, 32, 33, and *36*), fifteen genes related to photosystem II, five genes encoding photosystem I components, and six genes encoding ATP synthase and electron transport chain components (*atp*A, B, E, F, H, and I; Table 3).

The total GC content was 35.9% in cp genomes of the *P. juliflora*, *P. cineraria*, *P. glandulosa* and *P. javanica*, whereas the *A. microsperma* comprised 36.6%. The IR region shows the highest amount of GC contents (42.7%) compared to LSC (33%) and SSC (30.4%) regions. In these cp genomes the most frequently used codons were ATT (*n* = 1114, *n* = 1118) followed by AAA (*n* = 1181, *n* = 1117) in *P*. *juliflora* and *P. cineraria* respectively, which encodes isoleucine and lysine respectively. The least frequently used codon were ATT and ATC (*n* = 1) encode methionine in both *P. juliflora* and *P. cineraria* cp genomes. The AT contents of the 1st, 2nd, and 3rd codon positions of Protein Coding Sequences (CDSs) in both *P. juliflora* and *P. cineraria* were 64%; 64.5%, 63.9%; 63.6%, and 64.3%; 64.3% respectively (Table 4). These high AT contents detected here are similar to previously reported cp genomes of other terrestrial plants [28,36,37].

Protein-coding regions accounted for 48.4% and 48.1% of the *P. juliflora* and *P. cineraria* cp genomes respectively whereas the tRNA and rRNA regions accounted for 1.79%, 1.74% and 5.53%, 5.54% respectively. The remaining regions were intergenic spacers, introns, and non-coding sequences. The GC content counted 35.9% for both cp genomes, which is very similar to the previously reported cp genome of angiosperms [38,39].

### 2.2. SSR Analysis and Repeats, an Insight into the Genome

**Figure 2:**
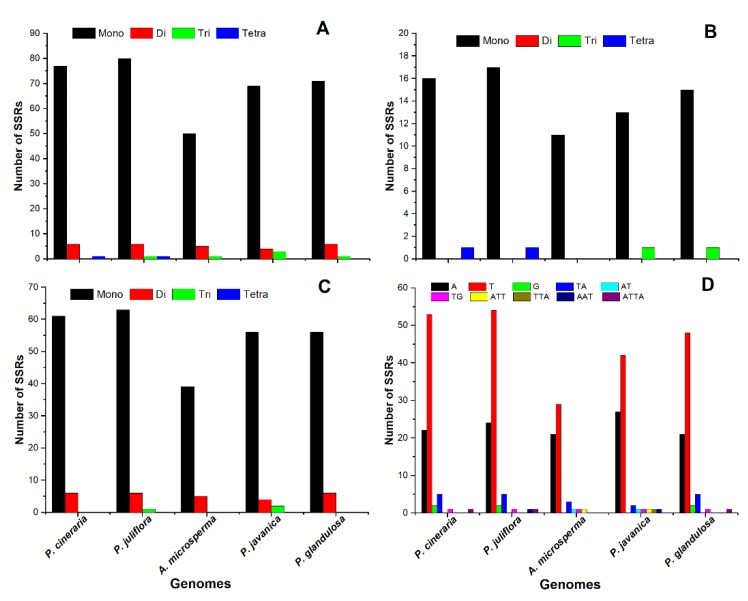
Analysis of simple sequence repeats (SSRs) in the *P. juliflora* and *P. cineraria* and related cp genomes. Total numbers of SSRs in whole genome (**A**), Number of SSRs in coding region (**B**), Number of SSRs in intergenic region (**C**) and Frequency of identified SSR motifs in different repeat class types (**D**).

We also determined SSRs in these cp genomes, which are commonly works as genetic markers in population genetics and evolutionary studies. SSRs or microsatellite markers, comprise of a sequence of 1–6 bp repeat units. In current study, SSRs were analyzed in three *Prosopis* species cp genomes as well as in two other cp genomes from the subfamily Caesalpinioideae. The total number of SSRs per species ranging from 56 to 88 and these repeats include 50–80 mononucleotide repeats, 4–6 dinucleotide repeats, 1–3 trinucleotide repeats, and one tetranucleotide repeats was only observed in *P. juliflora* cp genome. The maximum number of SSRs were detected in *P. juliflora* (88 SSRs), while the minimum number of SSRs was detected in *A. microsperma* (56 SSRs) (Figure 2). Mononucleotide repeats were found to be the most common types of SSRs in these cp genomes *P. juliflora*, *P. cineraria*, *A. microsperma*, *P. javanica*, *P. glandulosa*, comprising 90%, 91.6%, 89.2%, 90.7%, and 91% of total SSRs respectively (Figure 2). In *P. juliflora* the highest number of SSRs were single-base repeats (80), followed by double-base (6), and tri-base repeats (1) (Figure 2). Similarly, in *P. cineraria* the highest number were single base repeat (77), followed by double base repeat (6). However, among these cp genomes, only one tetra-base repeat was found only in *P. juliflora* cp genome.

In *P. juliflora* and *P. cineraria* most of the SSRs were detected in intergenic spacer (IGS) regions (79.5% and 79.7%) respectively, followed by coding sequences (CDSs) (20.4% and 10.2%) (Figure 2B,C). Mononucleotide A/T repeat units contained the highest proportion up to 90% in *P. juliflora*, 91.6% in *P. cineraria*, 91% in *P. glandulosa*, 90.7% in *P. javanica*, and 89.2% in *A. microsperma* (Figure 2D). Mononucleotide G repeats detected only in *P. juliflora, P. cineraria* and *P. glandulosa*. Among dinucleotides repeat, TA was found more frequently than AT. The tetranucleotide repeats were ATTA, which appeared only in *P. juliflroa* and *P. cineraria*, respectively (Figure 2D).

**Figure 3:**
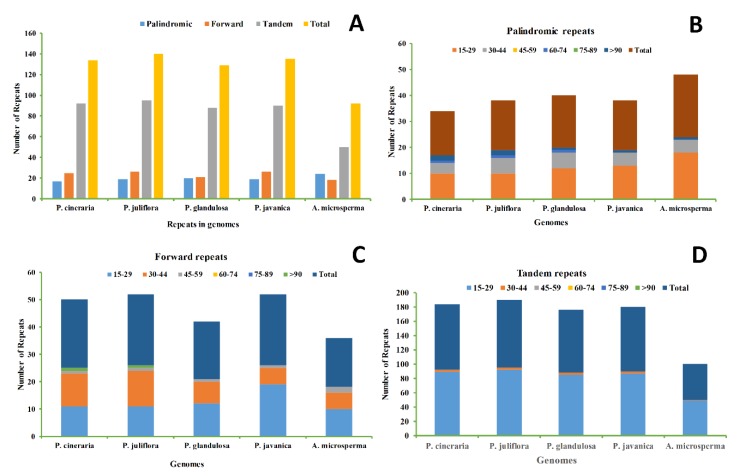
Analysis of repeated sequences in *P. juliflora* and *P. cineraria* and related cp genomes. Total numbers of the three repeat types (**A**), frequencies of palindromic repeats by length (**B**), frequencies of forward repeats by length (**C**) and frequencies of tandem repeats by length (**D**).

A total of 140, 134, 129, 135, and 92 repeats were detected in the *P. juliflora, P. cineraria, P. glandulosa, P. javanica,* and *A. microsperma* cp genomes, respectively. The *P. juliflora* genome comprises 26 forward, 19 palindromic, and 95 tandem repeats, while *P. cineraria* cp genome comprises 25 forward, 17 palindromic, and 92 tandem repeats, and about 21 forward, 20 palindromic and 88 tandem repeats were detected in *P*. *glandulosa* cp genome (Figure 3). Similarly, about 135 and 92 total repeats were also identified in related cp genome of both *P. javanica* and *A. microsperma,* respectively (Figure 3). With 24 palindromic repeats, *A. microsperma* comprises the maximum number of palindromic repeats, while *P. juliflora* and *P. javanica* comprises the highest number of forward repeats (26), and the highest tandem repeat was detected in *P*. *juliflora* (95). We also observed that *P. cineraria* comprises the minimum number of palindromic repeats (17) while *A. microsperma* comprises minimum number of forward repeats (18) and tandem repeats (50) (Figure 3).

### 2.3. Comparative Analysis and Sequence Divergence Analyses

**Figure 4:**
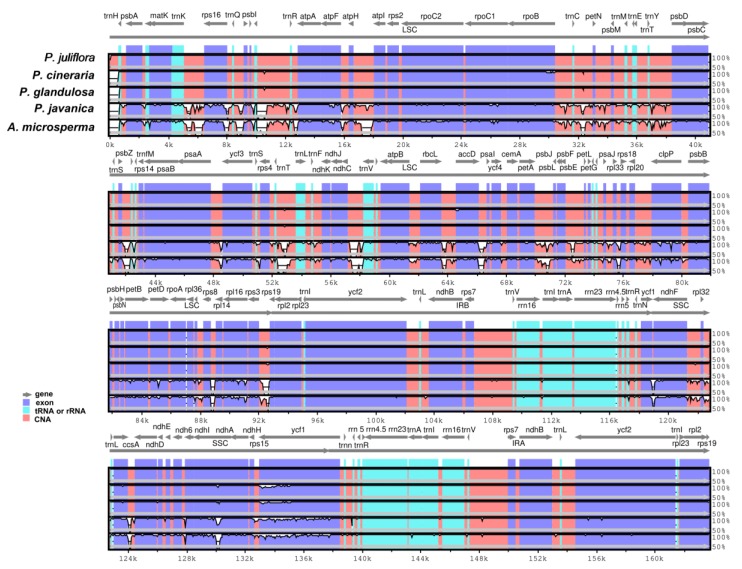
Visual alignment of plastid genomes from *P. juliflora* with *P. cineraria, P*. *glandulosa, P. javanica* and *A. micosperma* cp genomes. VISTA-based identity plot showing sequence identities among five species, using *P. juliflora* as a reference. Genome regions are color-coded as protein coding, rRNA coding, tRNA coding, or conserved noncoding sequences (CNS). The *x*-axis represents the coordinate in the chloroplast genome. Annotated genes are displayed along the top. The sequences similarity of the aligned regions is shown as horizontal bars indicating the average percent identity between 50% and 100%.

The *Prosopis* species cp genomes comparisons revealed various regions of sequence variation by using mVISTA and the *P. juliflora* genome was selected as reference genome. Some genes, such as *rpo*B*, acc*D*, ycf*1*, ccs*A*, atp*F*,* showed sequence divergence with *P. cineraria* and *P. glandulosa.* However, with *P. javanica* and *A. microsperma* it shows sequence divergence in many coding and non-coding regions such as *mat*K- *rps*16, psbI-*trn*R, *atp*H-*atp*I, *psb*Z-*trn*G, *rps*4-*trn*I, *pet*A-*psb*L, *rps*3-*rps*19, *ndh*G-*ndh*A, *rpoC*2, *rpo*B, *clp*P, *ndh*F, *ycf*1, and *mat*K exhibited sequence divergences among these genomes. *ycf*1, *ndh*H and *ndh*D were detected the most divergent genes in the SSC region. In the LSC region, the *rpoC*1*, rpoC*2*,* and *rpl*16 genes showed some sequence divergence only in *P*. *javanica* and *A*. *microsperma*. The IR region is very similar, however very little divergence was observed in *ycf*2 gene among the compared genomes (Figure 4).

**Figure 5:**
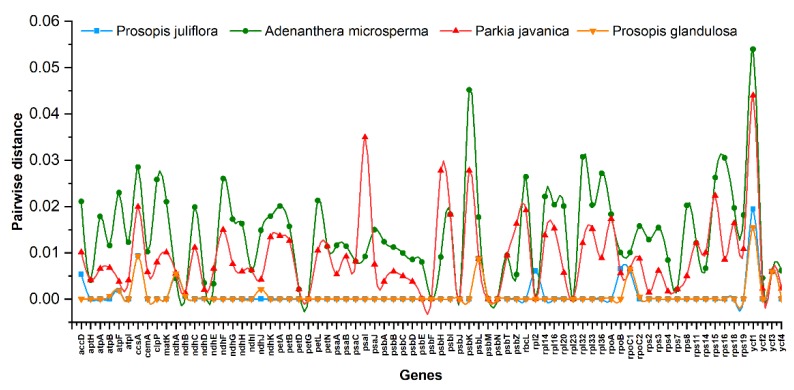
Pairwise distance of 75 genes from *P. cineraria* (as reference genome) with *P. juliflora*, *P. glandulosa*, *P. javanica* and *A. microsperma* cp genomes.

Similarly, we determined the average pairwise sequence divergence among these cp genomes (Supplementary Table S1). The *P. juliflora* cp genome revealed an average sequence divergence of 0.001, whereas the *P. juliflora* possessed the highest sequence divergence with *A. microsperma* (0.025) while the lowest was observed with *P. glandulosa* (0.0009). Furthermore, the thirteen most divergent genes among these genomes were *ycf*1, *psb*K, *psa*I, *rpl*32, *acc*D, *ccs*A, *clp*P, *ndh*F, *ndh*G, *psb*H, *rbc*L, *rps*15, and *rps*16. The *ycf*1 gene showed the highest average sequence divergence (0.1329), followed by *psb*K (0.072), *ccs*A (0.067), *rbc*L (0.045), and *acc*D (0.036; Figure 5). Similarly, among *Prosopis* species, *P. cineraria* showed greatest divergence in *ycf*1 with both *P. juliflora* (0.019) and *P. glandulosa* (0.015), respectively (Figure 5).

### 2.4. Boundaries between Inverted Repeat and Single Copy Regions

**Figure 6:**
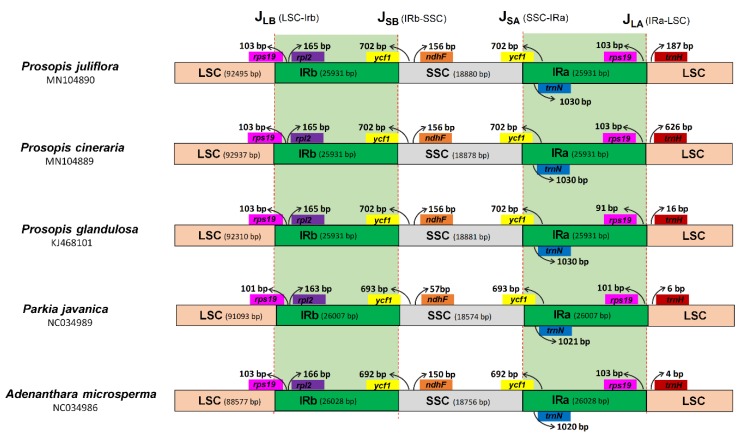
Distances between adjacent genes and junctions of the small single-copy (SSC), large single-copy (LSC), and two inverted repeat (IR) regions among five cp genomes within the subfamily Caesalpinioideae. Boxes above and below the primary line indicate the adjacent border genes. The figure is not to scale with regards to sequence length and only shows relative changes at or near the IR/SC borders.

In angiosperms, variation in the length of cp genomes are usually due to the IR and single-copy (SC) regions expansion and contraction [40]. In the current study, a comprehensive assessment of the four junctions (JLA, JLB, JSA, and JSB) between the two single copy regions (LSC and SSC) and the two IR regions (IRa and IRb) of the *P. juliflora, P. cineraria, P. glandulosa, P. javanica,* and *A. microsperma* cp genomes was performed. Regardless of the similar lengths of the IR regions of *P. cineraria, P. juliflora* and *P. glandulosa*, some expansion and contraction were detected, with the IR regions ranging from 25,931 bp in *Prosopis* species to 26,028 bp in *A. microsperma*. All the four junctions (JLA, JLB, JSA, and JSB) were conserved in three *Prosopis* cp genomes. However, some variations were observed with *P. javanica* and *A. microsperma* cp genomes (Figure 6). The partially duplicated genes were observed only at the beginnings and ends of the IR regions, including 103 bp of *rps*19 in *P. juliflora* and *P. cineraria* and 101 bp of r*ps*19 in *P. glandulosa* from J_LB_ (Figure 6). Furthermore, the *ycf*1 gene was partially duplicated, with 702 bp of this sequence being duplicated in *P. cineraria, P. juliflora, P. glandulosa,* 693 bp in *P. javanica,* and 692 bp in *A. microsperma.* Furthermore, *J_LA_* was found between *rps*19 and *trn*H, and the distance between *rps*19 and J_LA_ was 103 bp in *P. juliflora* and *P. cineraria* while in *P. glandulosa* this distance was 91 bp. However, in *P. javanica* and *A. micosperma* the above distance was 101 bp and 103 bp respectively. Additionally, variation was observed in the distance between J_LA_ and *trn*H among these species cp genomes (Figure 6). In *P. juliflora*, *P. cineraria,* and *P. glandulosa* the distance between JLA and *trn*H was 187 bp, 626 bp and 16 bp respectively. Similarly, this distance was 6 bp and 4 bp in *P. javanica* and *A. microsperma* cp genomes. The distance between J_SB_ border and *ndh*F gene was found to be the same in all *Prosopis* species cp genomes, while in *P. javanica* and *A. microsperma,* this distance was found 57 bp and 150 bp respectively (Figure 6).

### 2.5. Phylogenetic Relationships

**Figure 7:**
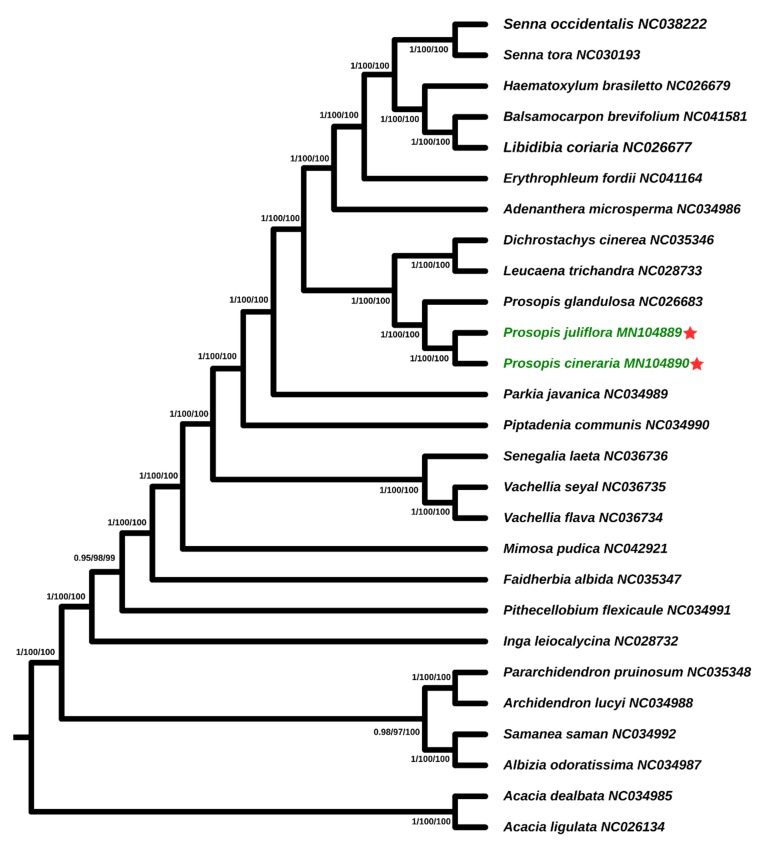
Phylogenetic tree of *P. juliflora* and *P. cineraria* within the subfamily Caesalpinioideae (Leguminosae). The entire genome data set was analysed using three different methods: Bayesian inference (BI), maximum parsimony (MP) and maximum likelihood (ML). Numbers above and below the branches represent bootstrap values in the MP and ML trees and posterior probabilities in the BI trees. The green color and red stars represent the positions of *P. juliflora* and *P. cineraria*.

The phylogenetic relationship of *P*. *cineraria, P*. *juliflora*, and *P*. *glandulosa* were determined within the subfamily Caesalpinioideae (Leguminosae) using 24 complete chloroplast genomes (Figure 7). Phylogenetic analysis using maximum likelihood (ML), maximum parsimony (MP), and Bayesian inference (BI) methods were performed. Our phylogenetic analysis for the species of subfamily Caesalpinioideae shows that *P*. *juliflora* and *P*. *cineraria* share monophyletic clade within the phylogenetic tree are supported by high bootstrap values in these two species, and further share a sub-clade with *P. glandulosa*. In addition, this study also revealed that, within the subfamily Caesalpinioideae, the genus *Prosopis* species are monophyletic and closely related to *Leucaena trichandra* and *Dichrostachys cinerea* (Figure 7).

## 3. Discussion

We sequenced the cp genomes of *P. juliflora* and *P. cineraria* using Ion Torrent S5 sequencing methods and compared them with those available for other species within subfamily Caesalpinioideae. The cp genomes was ranged from 115 to 165 kb in length have a circular structure, which comprised two copies of inverted repeat (IR) regions, a small single copy (SSC) region and a large single copy (LSC) region [41]. The cp genomes studied here were highly conserved, with genome sizes ranging from 159,389 bp in *A*. *microsperma* to 163,677 bp in *P. cineraria*, which encoded 128–131 genes (131 in *P. cineraria*, 132 in *P. juliflora*, 128 in *P. glandulosa*, 128 in *A. microsperma* and 130 in *P. javanica*) (Figure 1). The size range of these sequenced cp genomes are found similar with the sizes of the earlier reported cp genomes of *P. glandulosa* (163,040 bp) and related species [42,43,44]. Similarly, the IRs of these species are almost 26 kb in length and found similar and within the size range of typical angiosperm cp genomes (20 ± 28 kb) [45]. The difference in genome size could mainly be attributed to variation in the LSC regions rather than the expansion and contraction of IR region (Table 1) as reported previously [46]. Like other reported cp genome from Caesalpinioideae about 19 genes are duplicated in the IR regions (Figure 1 and Table 1), including four rRNA genes, seven tRNA genes, and eight protein coding genes [42,44]. Furthermore, eighteen genes (twelve protein coding genes and six tRNA genes) having introns were detected in these genomes and among these introns containing genes *rps*12*, clp*P and *ycf*3 genes have two introns each (Table 2). Among these genes, *rps12* was unevenly divided and its 5′ exon is detected in the LSC region and one copy of the 3′ exon and intron are detected in each of the IR regions as reported previously in other angiosperms. Contrast to *P. glandulosa* cp genome *trnG-GCC* was absent in both *P. cineraria* and *P. juliflora* cp genomes while in other two cp genome of *A. microsperma* and *P. javanica, trnG-GCC* gene was present without intron. Like other plant species from family Caesalpinioideae, the maturase K (*mat*K) gene is annotated within the *trn*K intron [31]. The GC content of the *P. juliflora* and *P. ceneraria* LSC, SSC, and IR region were 33.1%, 30.4%, and 42.7%, respectively. Due to the existence of eight ribosomal RNA (rRNA) in IR regions higher GC contents were observed in these regions like other angiosperm cp genomes [42,47].

Repetitive sequences play significant roles in rearrangement and stabilization and cp genome sequences [48] and can affect copy number difference among similar and different species. Length variation and variable copy numbers have encouraged the extensive use of cp SSRs in biogeographic studies and plant population genetics, particularly at lower taxonomic levels [49,50]. These characteristics in cp genomes can be used for molecular marker designing and play vital role in plant identification [51] and phylogenetic analyses [52]. A total of 140, 134, and 129 repeats were found in the *P. juliflora, P. cineraria,* and *P. glandulosa* cp genomes, respectively. Cp genome repeat sequences contribute significantly to genomic structural variations, rearrangements or expansions [41,53]. Similarly, about 135 and 92 total repeats were detected in the *P. javanica* and *A. microsperma* cp genomes, respectively (Figure 2). *P. juliflora* comprises the lowest number of forward repeats (19) while the lowest palindromic repeats were found in *P. cineraria* cp genome (Figure 3). In our study, tandem repeats were determined to be the most plentiful in the *P. juliflora* (95) cp genome, showing similar traits to the previously reported cp genome [42,44]. It is obvious from earlier reports that high number and complex repeats also play key roles in cp genomes rearrangement and evolutions [53,54,55].

Simple sequence repeats (SSRs) characterize potentially valuable markers because of relative lack of recombination, their haploid nature, and maternal inheritance for phylogenetic studies [56]. SSRs have been extensively used in the estimation of levels of genetic variation, analyzing gene flow, describing the history of populations in plants and animals [57,58]. The efficacy of the SSR markers in genetic screening has been reported in other *Prosopis* species such as *P. chilensis* (Mol.) Stuntz, *P. alba* Griseb., *P. flexuosa* D.C., *P. juliflora* Swartz DC., *P. pallida* Humbolt & Bonpland ex Willd., *P. rubriflora* Hassl., and *P. ruscifolia* Griseb [38,39,59,60,61]. We analyzed the type and distribution of SSRs in the *P. juliflora* and *P. cineraria* with related species cp genomes and detected the highest number of SSRs in *P. juliflora* (88) followed by 84 SSRs, including 13 compound SSRs in each (Figure 3). The detection of AT-rich SSRs in *P. cineraria* and *P. juliflora* cp genomes were similar other plant species [56]. According to Ebert and Peakall [56], intra-species variation in cp genomes are due to mononucleotide cpSSRs present in a non-coding single copy (SC) region. The observed results accord with previous findings that cp genomes SSRs are usually comprised of polyadenine (polyA) or polythymine (polyT) repeats and occasionally contain tandem guanine (G) or cytosine (C) repeats [62], thereby contributing to AT richness of cp genomes [39,40].

Like previously reported angiosperm cp genome the IR regions showed lower sequence divergence compared to SSC and LSC regions. Moreover, pairwise alignment of the *P. juliflora* cp genome with those of four other genomes displayed a high degree of synteny. Similarly, relatively lower sequence identity was observed among these cp genomes, especially in the *mat*K-*rps*16, *psb*I-*trn*R, *atp*H-*atp*I, *psb*Z-*trn*G, *rps*4-*trn*I, *pet*A-*psb*L, *rps*3-*rps*19, *ndh*G-*ndh*A, *rpoC*2, *rpo*B, *clp*P, *ndh*F, *ycf*1 and *mat*K regions (Figure 4). In addition, the SSC and LSC regions showed more divergence than the two inverted repeat regions in all *Prosopis* cp genomes, and the non-coding regions were less similar than the coding regions as reported previously [28,63]. The current results also revealed similar variations among numerous coding regions in these cp genomes, as suggested by Kumar et al. [64]. Moreover, numerous researchers have analyzed coding and non-coding regions especially having high variability as possible molecular markers family Fabaceae, such as *rpl*16*-rps*3, *trn*S(GGA)-*trn*G(UCC), *atp*B*-rbc*L and *trn*T*-trn*L [65,66]. Similarly, it has been reported that coding regions in cp genome reveal less variability than non-coding regions and therefore, these non-coding regions became a key region to infer the phylogenetic position in various species [28,67].

Similarly, the average pairwise sequence divergence among the cp genomes of the *Prosopis* and related species were calculated (Supplementary Table S1). The cp genome of *P. juliflora* showed an average sequence divergence of 0.001, whereas the *P. juliflora* possessed the highest sequence divergence with *A. microsperma* (0.025) while the lowest was observed with *P. glandulosa* (0.0009). Furthermore, the *ycf*1 gene revealed the highest average sequence divergence (0.1329), followed by *psb*K (0.072), *ccs*A (0.067), *rbc*L (0.045), and *acc*D (0.036; Figure 5). Similarly, among *Prosopis* species the *P. cineraria* showed greatest divergence in *ycf*1 with both *P. juliflora* (0.019) and *P. glandulosa* (0.015) respectively.

In spite of the collinear gene order found in most land plant, some notable changes such as gene loss [68], sequence inversion [69], and contraction and expansion at the borders between IRs, SSC, and LSC regions [70]. Similarly, length variation among cp genomes were observed due to the contraction and expansion of the IR regions [71,72]. However, IR regions have been lost in some cp genomes, such as *Erodium, Carnegiea* [73], and some Fabaceae members [74]. The *Prosopis* cp genomes were highly conservative in size, structure, SC and IR boundary locations among species were slightly diverse due to contraction or expansion of the cp genome, as reported in most land plants [75,76,77]. In the current study a comprehensive assessment of the four junctions (JLA, JLB, JSA, and JSB) between the two single copy regions (LSC and SSC) and the two IR regions (IRa and IRb) of the *P. cineraria, P. juliflora, P. glandulosa, P. javanica,* and *A. microsperma* cp genomes was performed. Regardless of the parallel lengths of the IR regions of *P. cineraria, P. juliflora*, and *P. glandulosa*, some extension and contraction were detected, with the IR regions ranging from 25,931 bp in *Prosopis* species to 26,028 bp in *A. microsperma*. All the four junctions (JLA, JLB, JSA, and JSB) were conserved in three *Prosopis* cp genomes. However, some variations were observed with *P. javanica* and *A. microsperma* cp genomes (Figure 6). The partially duplicated genes found at the beginnings and ends of the IR regions, including 103 bp of *rps*19 in *P. juliflora* and *P. cineraria* and 101 bp of *rps*19 in *P. glandulosa* from J_LB_. The boundaries between IRs, SSC, and LSC were similar in all the cp genomes studied. The IRb/LSC boundary of the studied cp genomes from subfamily Caesalpinioideae is detected in the *rps*19 gene, and a small portion of the *rps*19 gene is also found in the IRb region, as reported in previously angiosperm cp genome such as in *O. vulgare* [78], *S. miltiorrhiza*, and some species from *Ilex* genus [79]. On the other hand, some cp genomes such as in *Lupinus luteus* [80] and *Millettia pinnata* [81] the *rps*19 gene does not extend into the IR region. Similar results were mostly reported in numerous monocots cp genome such as, in the *Oryza* AA genome [82], the *rps*19 gene present inside the IR region [83].

Cp genomes have been valuable in molecular, evolutionary, and phylogenetic studies. Numerous analyses on the basis of complete genome sequence comparison [28,84] have resolved various phylogenetic problems at deep node levels and improved our understanding of mysterious evolutionary associations among angiosperms. The phylogenetic relationships of *P*. *cineraria, P*. *juliflora*, and *P*. *glandulosa* were determined within the subfamily Caesalpinioideae (Leguminosae) using complete chloroplast genomes from 24 plant cp genomes (Figure 7). Despite of numerous analyses of relationship within the subfamily Caesalpinioideae its evolutionary history remains poorly understood [85,86,87,88,89]. As reported recently by Lewis et al. [90] subfamily Caesalpinioideae is paraphyletic; it comprises the monophyletic tribes Detarieae and Cercideae, and the paraphyletic tribes Caesalpinieae and Cassieae. Phylogenetic analysis using maximum likelihood (ML), maximum parsimony (MP), and Bayesian inference (BI) methods were performed. Our phylogenetic analysis for the species of subfamily Caesalpinioideae shows that *P*. *juliflora* is closer to P. *cineraria* than *P. glandulosa* with high bootstrap support. In addition, this study also revealed that, within the subfamily Caesalpinioideae, the genus *Prosopis* species are monophyletic and closely related to *Leucaena trichandra* and *Dichrostachys cinerea* (Figure 7).

## 4. Methodology

### 4.1. Sample Collection

The fresh young leaves of *P. juliflora* and *P. cineraria* were collected from plants growing in Nizwa Oman (22°59′48.8″ N; 57°39′48.7″ E). The Director General of Nature Conservation, Ministry of Environment & Climate Affairs, Sultanate of Oman had issued a collection permit (4/2106) for this purpose. The plants were identified by Taxonomist at Royal Botanical Garden, Oman. The area receives limited rainfall throughout the year, having an average temperature from 25 °C to 46 °C in summer season. The leaf samples were collected in plastic zip bags and kept for DNA extraction in liquid nitrogen at −80 °C.

### 4.2. DNA Extraction and Genome Sequencing

Chloroplast DNA was extracted from the powdered leaves of *P. juliflora* and *P. cineraria* by following a modified protocol [91]. Manufacturer’s instructions (Life Technologies USA, Eugene, OR, USA) were followed to prepare genomic libraries. Ion Shear™ Plus Reagents kit was used to share the cpDNA into 400 bp fragments enzymatically and Ion Xpress™ Plus gDNA Fragment Library kit was used to prepare libraries. Qubit 3.0 fluorometer and bioanalyzer (Agilent 2100 Bioanalyzer system, Life Technologies USA) were used to quantify and qualify libraries. Ion OneTouch™ 2 instrument was used to amplify template after library preparation and the amplified templates were enriched (Ion OneTouch™ ES enrichment system) by using Ion 530 & 520 OT2 Reagents. Ion S5 sequencing protocol was used to load the samples on Ion S5 530 Chip for sequencing.

### 4.3. Chloroplast Reference-Based Genome Assembly

A total of 1,126,428 and 1,238,421 raw reads were produced for *P. cineraria* and *P. juliflora*, respectively. The generated both cp genomes reads were mapped to *P. glandulosa* (KJ468101) which is used as reference genome using Bowtie2 (v.2.2.3) [92] in Geneious Pro (v.10.2.3) software [93]. The *P. juliflora* and *P. cineraria* assemblies mean coverage were 213X and 175X respectively. The previously published genome of *P. glandulosa* was used to identify the IR junctions using MITObim (v.1.8) software [94].

### 4.4. Prosopis Species Genome Annotation

For both *P. cineraria* and *P. juliflora* cp genomes annotation Dual Organellar Genome Annotator (DOGMA) [95] was used using BLASTN and BLASTX to determine the locations of tRNA, ribosomal RNAs and protein coding genes, and tRNAscan-SE version 1.21 [96] software was used to detect tRNA genes. Moreover, for manual alteration, tRNAscan-SE [96] and Geneious Pro (v.10.2.3) [93] were used to compare the genomes with the already reported *P. glandulosa* genome and intron boundaries, start and stop codons were also adjusted manually. Similarly, the *Prosopis* species cp genomes structural features were illustrated using OGDRAW [97]. Furthermore, for genome divergence among these species, cp genomes mVISTA [98] in Shuffle-LAGAN mode was used and *P. juliflora* was selected as reference genome.

### 4.5. Repeat Identification

For identification of forward and reverse repeats REPuter software [99] was used. About 15 bp sequence with 90% identities was considered a minimum criterion. Moreover, MISA software [100] was used to determine SSRs with following search parameters: ≥10 repeat units for single base pair repeats; ≥8 repeat units for two base pair repeats; ≥4 repeat units for three and four base pair repeats; and ≥3 repeat units for five and six base pair repeats,. To find the tandem repeats, Tandem Repeats Finder version 4.07 [101] with default settings was used.

### 4.6. Chloroplast Genome Divergence and Phylogenetic Relationship

The whole genome and shared genes sequence divergence among *Prosopis* species and related species were calculated. Comparative analysis strategy was used after multiple sequence alignment and comparing gene order to identify the ambiguous and missing gene annotation. To align the complete cp genomes, MAFFT version 7.222 [102], with default parameters, was used and Kimura’s two-parameter (K2P) model [103] was used to determine pairwise sequence divergence. To infer the phylogenetic positions of *P. cineraria* and *P. juliflora* within the sub family Caesalpinioideae (Leguminosae), 23 cp genome sequences were obtained from the NCBI database. Alignments of the complete cp genomes were constructed on the bases of conserved gene orders and the structures of the cp genomes [41], and three different methods were applied to infer phylogenetic tree: Bayesian inference (BI), implemented in Mr Bayes 3.1.2 [104], maximum parsimony (MP) using PAUP 4.0 [105], and maximum likelihood (ML) using MEGA 6 [106], employing previously described settings [36,40]. The best substitution model GTR + G was tested by jModelTest version v2.1.02 [107] according to the Akaike information criterion (AIC) for Bayesian posterior probabilities (PP) in BI analyses. The Markov Chain Monte Carlo (MCMC) method was run using four incrementally heated chains across 1,000,000 generations, starting from random trees and sampling 1 out of every 100 generations. The values of first 30% of trees were discarded as burn-in. Maximum parsimony run was based on a heuristic search with 1000 random addition of sequence replicates with the tree-bisection-reconnection (TBR) branch-swapping tree search criterion to estimate the posterior probabilities. Similarly, the parameters for ML analysis were optimized using a BIONJ tree [108] as the starting tree with 1000 bootstrap replicates by employing the Kimura 2-parameter model with invariant sites and gamma-distributed rate heterogeneity

## 5. Conclusions

In the present study, the *P*. *juliflora* and *P*. *cineraria* complete genomes sequences were determined. The genomics characteristics genome size, GC contents, genome organization and gene orders were found to be highly conserved as compared to related cp genomes. Repetitive sequences such as tandem repeats and SSRs were analyzed within these cp genomes. The maximum number of simple sequence repeats was detected in *P*. *juliflora*, followed by *P*. *cineraria* and *P. glandulosa*. Overall, a high degree of sequence similarity between *P. cineraria* and *P. glandulosa* was observed. However, various divergent genes, such as *rpo*B, *acc*D, *ycf*1, *ccs*A, and *atp*F, were found in these cp genomes. The present study provides a valuable set of complete chloroplast genome analysis of *P. juliflora* and related species, which could be helpful for species identification and may facilitate biological, genetic diversity, and phylogenetic studies.

## Figures and Tables

**Table 1:** Summary of genome features of complete chloroplast of *P. cineraria*, *P. juliflora*, *P. glandulosa*, *A. microsperma*, *P. javanica*.

	*P. Cineraria*	*P. Juliflora*	*P. Glandulosa*	*A. Microsperma*	*P. Javanica*
Size (bp)	163,677	163,237	163,040	159,389	161,681
Overall GC contents	35.9	35.9	35.9	36.5	35.9
LSC size (bp)	92,937	92,495	92,310	88,577	91,093
SSC size (bp)	18,878	18,880	19,132	18,756	18,574
IR size (bp)	25,931	25,931	25,931	26,028	26,007
Protein coding regions (bp)	78,883	78,421	78,039	78,030	78,075
tRNA size (bp)	2868	2927	2810	2793	2794
rRNA size (bp)	9052	9052	9052	9052	9052
Number of genes	131	132	128	128	130
Number of protein coding genes	85	85	83	83	83
Number of rRNAs	8	8	8	8	8
Number of tRNA s	38	39	37	37	37
Genes with introns	21	21	23	22	23

P. cineraria = Prosopis cineraria, P. juliflora = Prosopis juliflora, P. glandulosa = Prosopis glandulosa, A. microsperma = Adenanthera microsperma, P. javanica = Parkia javanica.

**Table 2:** The genes with introns in the three *Prosopis* species chloroplast genomes and the length of exons and introns.

Gene	Location	Exon I (bp)			Intron 1 (bp)			Exon II (bp)			Intron II (bp)			Exon III (bp)		
		P. j	P. c	P. g	P. j	P. c	P. g	P. j	P. c	P. g	P. j	P. c	P. g	P. j	P. c	P. g
*atp*F	LSC	145	145	145	744	726	727	389	407	407						
*pet*B	LSC	6	6	6	815	815	815	642	642	642						
*pet*D	LSC	8	8	8	720	720	720	475	475	475						
*rpl*2 ^a^	IR	391	393	393	665	662	662	434	435	435						
*rpl*16	LSC	9	9	9	1173	1173	1173	399	399	399						
*rps*16	LSC	40	40	40	884	883	883	245	245	245						
*rpo*C1	LSC	432	432	432	842	802	802	1578	1617	1617						
*rps*12 *	LSC-IR	114	114	114				232	232	232	540	540	540	26	26	
*clp*P	LSC	69	69	69	789	790	789	291	291	291	642	642	642	228	228	228
*ndh*A	SSC	552	553	552	1449	1452	1457	540	539	540						
*ndh*B ^a^	IR	777	777	777	685	684	685	756	756	756						
*ycf*3	LSC	126	126	126	729	729	728	228	228	228	738	738	738	153	153	153
*trn*A-UGC ^a^	IR	38	38	38	802	794	802	35	35	35						
*trn*I –GAU^a^	IR	42	42	42	948	948	948	35	35	35						
*trn*L-UAA	LSC	37	37	37	536	536	536	50	50	50						
*trn*K-UUU	LSC	29	29	29	2492	2491	2491	37	37	37						
*trn*G-GCC	LSC			23			702			49						
*trn*V-UAC	LSC	37	37	37	619	619	619	39	39	39						

P. j = *Prosopis juliflora*, P. c = *Prosopis cineraria*, P. g = *Prosopis glandulosa*. ^a^ Duplicated gene. * The *rps*12 coding sequence is split between 5*′-rps*12 and 3*′-rps*12, which are located in the large single-copy region and inverted repeat region, respectively.

**Table 3:** Genes in the sequenced *Prosopis* species chloroplast genomes.

Category	Group of Genes	Name of Genes
**Self-replication**	Large subunit of ribosomal proteins	*rpl*2, 14, 16, 20, 23, 32, 33, 36
	Small subunit of ribosomal proteins	*rps*2, 3, 4, 7, 8, 11, 12, 14, 15, 16, 18, 19
	DNA dependent RNA polymerase	*rpo*A*,* B*, C*1*, C*2
	rRNA genes	*rrn*4.5*, rrn*5*, rrn*16*, rrn*23
	tRNA genes	*trn*A-UGC, *trn*C-GCA, *trn*D-GUC, *trn*E-UUC *trn*F-GAA, *trn*fM-CAU, *trn*G-UCC, *trn*H-GUG, *trn*I-CAU, *trn*I-GAU, *trn*K-UUU, *trn*L-CAA, *trn*L-UAA, *trn*L-UAG, *trn*M-CAU, *trn*N-GUU, *trn*P-GGG, *trn*P-UGG, *trn*Q-UUG, *trn*R-ACG, *trn*R-UCU, *trn*S-GCU, *trn*S-GGA, *trn*S-UGA, *trn*T-GGU, *trn*T-UGU, *trn*V-GAC, *trn*V-UAC, *trn*W-CCA, *trn*Y-GUA
**Photosynthesis**	Photosystem I	*psa*A*,* B, C, I,
	Photosystem II	*psb*A*,* B, C, D, E, F, H, I, J, K, L, M, N, T, Z
	Cytochrome b6/f complex	*pet*A*,* B, D, G, L, N
	ATP synthase	*atp*A, B, E, F, H, I
	Rubisco	*rbc*L
	Maturase	*mat*K
	Protease	*clp*P
	Envelop membrane protein	*cem*A
	Subunit Acetyl- CoA-Carboxylate	*acc*D
	c-type cytochrome synthesis gene	*ccs*A
**Unknown**	Conserved Open reading frames	*ycf*1, 2, 3, 4

**Table 4:** Base composition of the *P. cineraria, P. juliflora* and *P. glandulosa* chloroplast genome.

	T/U			C			A			G			Length (bp)		
	P. j	P. c	P. g	P. j	P. c	P. g	P. j	P. c	P. g	P. j	P. c	P. g	P. j	P. c	P. g
Genome	32.5	32.5	32.5	18.3	18.3	18.3	31.6	31.6	31.7	17.6	17.6	17.6	163,237	163,677	163,040
LSC	34.2	34.2	34.2	17.0	17.0	17.0	32.6	32.6	32.6	16.1	16.2	16.1	92,495	92,937	92,310
SSC	34.9	34.9	34.6	15.9	16.0	15.7	34.7	34.6	35.2	14.5	14.5	14.4	18,880	18,878	19,132
IR	28.8	28.8	28.5	20.6	20.6	22.1	28.5	28.5	28.8	22.1	22.1	20.6	25,931	25,931	25,931
tRNA	25.1	24.9	24.8	23.4	23.5	23.8	22.1	22.0	21.9	29.4	29.6	29.5	2927	2868	2810
rRNA	18.9	18.9	18.8	23.7	23.7	23.6	25.7	25.7	25.7	31.7	31.7	31.9	9052	9052	9052
Protein coding genes	31.7	31.7	31.7	17.4	17.4	17.4	30.9	30.8	30.8	20.0	20.1	20.1	78,421	78,883	78,039
1st position	32.6	33.0	32.4	17.9	18.14	18.2	31.4	31.5	31.7	17.9	17.5	17.5	54,412	54,559	54,347
2nd position	32.4	32.33	32.7	18.5	18.4	17.7	31.5	31.3	32.1	17.4	17.8	17.5	54,412	54,559	54,347
3rd position	32.3	32.44	32.4	20.9	18.1	18.8	32.0	31.9	31	17.4	17.3	17.7	54,412	54,559	54,347

P. c = Prosopis cinerea, P. j = Prosopis juliflora, P. g = Prosopis glandulosa.

## Supplementary Materials

Supplementary materials can be found at https://www.mdpi.com/1422-0067/21/9/3280/s1.

## Abbreviations

BI	Bayesian inference
IR	Inverted repeat
LSC	Large single copy
ML	maximum likelihood
MP	Maximum parsimony
NGS	Next generation sequencing
rRNA	Ribosomal RNA
SSC	Short single copy
SSRs	Simple sequence repeats
tRNA	Transfer RNA
